# Pseudo–Messenger RNA: Phantoms of the Transcriptome

**DOI:** 10.1371/journal.pgen.0020023

**Published:** 2006-04-28

**Authors:** Martin C Frith, Laurens G Wilming, Alistair Forrest, Hideya Kawaji, Sin Lam Tan, Claes Wahlestedt, Vladimir B Bajic, Chikatoshi Kai, Jun Kawai, Piero Carninci, Yoshihide Hayashizaki, Timothy L Bailey, Lukasz Huminiecki

**Affiliations:** 1 Genome Exploration Research Group (Genome Network Project Core Group), RIKEN Genomic Sciences Center, RIKEN Yokohama Institute, Yokohama, Japan; 2 Institute for Molecular Bioscience, University of Queensland, Brisbane, Australia; 3 Wellcome Trust Sanger Institute, Hinxton, United Kingdom; 4 Institute for Infocomm Research, Singapore, Singapore; 5 University of the Western Cape, South African National Bioinformatics Institute, Bellville, South Africa; 6 Center for Genomics and Bioinformatics, Karolinska Institutet, Stockholm, Sweden; 7 Department of Biomedical Sciences, The Scripps Research Institute, Jupiter, Florida, United States of America; 8 Genome Science Laboratory, Discovery Research Institute, RIKEN Wako Institute, Wako, Japan; 9 Ludwig Institute for Cancer Research, Uppsala Universitet, Uppsala, Sweden; The Jackson Laboratory, US; MRC-Harwell, UK; NHGRI-NIH, US; Lawrence Livermore National Laboratory, US; The Jackson Laboratory, US

## Abstract

The mammalian transcriptome harbours shadowy entities that resist classification and analysis. In analogy with pseudogenes, we define pseudo–messenger RNA to be RNA molecules that resemble protein-coding mRNA, but cannot encode full-length proteins owing to disruptions of the reading frame. Using a rigorous computational pipeline, which rules out sequencing errors, we identify 10,679 pseudo–messenger RNAs (approximately half of which are transposon-associated) among the 102,801 FANTOM3 mouse cDNAs: just over 10% of the FANTOM3 transcriptome. These comprise not only transcribed pseudogenes, but also disrupted splice variants of otherwise protein-coding genes. Some may encode truncated proteins, only a minority of which appear subject to nonsense-mediated decay. The presence of an excess of transcripts whose only disruptions are opal stop codons suggests that there are more selenoproteins than currently estimated. We also describe compensatory frameshifts, where a segment of the gene has changed frame but remains translatable. In summary, we survey a large class of non-standard but potentially functional transcripts that are likely to encode genetic information and effect biological processes in novel ways. Many of these transcripts do not correspond cleanly to any identifiable object in the genome, implying fundamental limits to the goal of annotating all functional elements at the genome sequence level.

## Synopsis

Our understanding of genetics has been dominated by the so-called central dogma: the theory that DNA is transcribed into RNA, which is translated via the genetic code to produce proteins. Thus, DNA is the inherited store of genetic information, proteins are the end products that carry out cellular functions, and RNA is a kind of passive intermediary, hence termed messenger RNA. However, evidence has been accumulating that RNA plays a much more dynamic role than this. This study provides an unprejudiced survey of “pathological” RNA molecules, which resemble protein-coding RNA except that they contain violations of the genetic code. These pseudo–messenger RNAs constitute a surprisingly large fraction of all transcripts, as much as 10%. These ghostly molecules have always been present in RNA surveys, but have stayed below the radar because they do not cleanly correspond to annotated elements in DNA, i.e., “genes”. Their prevalence demonstrates that RNA is a distinct continent that cannot be fully understood as a mirror of DNA or proteins.

## Introduction

The transcriptome is a cosmopolitan community. While standard protein-coding messenger RNA is the most widely recognised type of transcript, other categories exist that have suffered scientific discrimination because they tend not to fit the traditional view of molecular biology. Isolated examples of non-protein-coding RNA have long been recognised, but recent evidence argues for a much larger number of noncoding transcript species [1–3]. However, there exist yet more mysterious transcripts that seem to be intermediate between coding and noncoding. These include transcribed pseudogenes, for which, again, isolated examples have been known for some time [4,5], and recent studies show more widespread transcription of pseudogenes [6–8]. There are also many variants of protein-coding genes with disrupted reading frames [9]: these have been dismissed as experimental noise, biological noise, or, at best, regulated splicing of unproductive transcripts as a form of gene regulation [10]. A final category are the recoded mRNAs, which encode proteins but violate the standard genetic code in various ways, e.g., using the opal stop codon to encode selenocysteine, or employing programmed ribosomal frameshifting or stop codon readthrough [11]. Our knowledge of these non-standard transcript classes has been limited because most experimental and computational gene detection projects are designed for standard protein-coding mRNA.

The FANTOM collection of more than 100,000 full-length mouse cDNA sequences offers a great opportunity to survey non-standard transcript categories [3]. Although the FANTOM annotation procedure is primarily focused on standard protein-coding mRNA, it became clear that the collection includes many transcripts that appear to encode proteins, but suffer disruptions to the reading frame. Some of these reflect sequencing errors or cloning artefacts, but not all. We propose the term pseudo–messenger RNA (ψmRNA) to describe such transcripts. This term is useful, as opposed to, say, transcribed pseudogene, because it is not always easy to tell whether a ψmRNA is in fact a transcribed pseudogene, or a disrupted variant of a protein-coding gene. More fundamentally, we suspect this may be a false dichotomy: recent evidence suggests that transcribed regions of the genome form interlaced networks rather than being separated into discrete “genes” [1,3], so there may be no clear answer as to which gene or pseudogene a transcript belongs to.

Pseudogenes are easy to recognise but hard to define. They are genomic sequences that resemble functional genes but are in some sense non-functional, although the definition of “non-functional” has proven slippery [8], especially given the existence of pseudogenes that clearly are functional [12]. Pseudogenes are classified as either processed or unprocessed. The former arise through retrotransposition of RNA sequences into the genome, and are recognisable by their lack of introns and by other features. It is often said that the latter arise by gene DNA duplication, so it is important to point out that they can also result from decay of formerly functional genes. For example, all mammals except primates and guinea pig can synthesise vitamin C, using an enzyme called l-gulono-gamma-lactone oxidase, which persists in humans as a vestigial pseudogene [13]. In any case, we might expect that processed pseudogenes are less likely than unprocessed pseudogenes to be transcribed, since retrotransposition does not duplicate the promoter.

The ψmRNAs that we identify here will inevitably include some recoded mRNAs, which are not really “pseudo” because they encode full-length proteins via non-standard translation rules. It would be logical to call them pseudo-ψmRNAs (and no doubt cases with indefinite further iterations of “pseudo” exist, too). Thus, our ψmRNA list offers a useful starting point for identifying recoded mRNA, and we present evidence that a few hundred of them actually encode selenoproteins. The available evidence suggests that recoded mRNA is rare and will only constitute a small fraction of our ψmRNA list, but historical biases against identifying recoded mRNA make this conclusion tentative [11].

Another potentially confounding phenomenon is RNA editing. Some mammalian mRNAs undergo adenosine-to-inosine editing, which occurs co-transcriptionally and prior to splicing, and a smaller number are known to undergo cytosine-to-uracil editing, which also takes place in the nucleus [14,15]. Our ψmRNA scan might pick up immature mRNAs with internal stop codons that are removed by editing, but since we study mature FANTOM transcripts and editing occurs early during RNA maturation, this should not be a common occurrence.

We submit that it is improper to dismiss the biological importance of ψmRNAs. Firstly, they may have a function through interactions with coding transcripts, influencing nuclear export, mRNA stability, splicing, or efficiency of translation. For example, it has been shown that the mouse expressed pseudogene Makorin1-pl regulates mRNA stability of its coding paralogue, and that it is conserved in nucleotide sequence [12,16]. Secondly, ψmRNAs may encode proteins that are truncated (premature stop codons) or partially scrambled (simple frameshifts and compensating frameshifts), and these are likely to function as regulators in hetero-dimeric complexes with proteins encoded by paralogues. This survey demonstrates that, although their biological functions are almost always unknown, ψmRNAs are real and numerous citizens of the transcriptome. Since they break the usual rules for encoding proteins, the implication is that they encode genetic information and effect biological processes in novel ways.

## Results

### Criteria for Identifying ψmRNAs

We identified ψmRNAs by aligning the FANTOM3 cDNA sequences against all known proteins (from all organisms) in the Swiss-Prot database [17], and retaining alignments with frameshifts and/or internal stop codons. The alignments were performed by the program FASTX, which translates the cDNA in all three frames, and allows alignments to switch between these frames with forward and reverse frameshifts [18]. FASTX also estimates the statistical significance of each alignment in terms of an *E*-value. These *E*-values are very accurate, unless the sequences have unusual monomer compositions [19]. Therefore we filtered low complexity and tandem repeat sequences using the programs PSEG and XNU. We retained alignments with *E* ≤ 0.01, meaning that spurious matches to unrelated proteins are expected for 1% of the cDNAs. For each cDNA, only the alignment to its closest Swiss-Prot homologue (top FASTX hit) was considered.

It is essential to demonstrate that these ψmRNAs are real biological transcripts rather than experimental artefacts. Reading frame disruptions often indicate sequencing errors in the cDNA. To exclude these cases, we required independent confirmation of disruptions from the mouse genome sequence. Taking the cDNA-to-genome alignments produced by the FANTOM3 Consortium, we checked that the protein-aligned region of cDNA aligned to the genome without any gaps, except for introns, defined as gaps in the cDNA only of size 15 nt or more and flanked by the standard splice sequences GT and AG. This criterion ensures that frameshifts within the protein–cDNA alignment have no corresponding gaps in the cDNA–genome alignment; so if the frameshifts are due to sequencing errors, the same error must be present in both the cDNA and the genome sequence, which is extremely unlikely. In addition, internal stop codons were counted only if they aligned to identical genomic sequences. Thus, these transcripts contain reading frame disruptions that are not caused by sequencing error.

Although these cDNAs are confirmed by the genome sequence, it might still be argued that they represent intronic or untranscribed sequences, from erroneous cloning of pre-mRNA or DNA. However, many of the ψmRNAs have exon–exon junctions within the protein-aligned region (Table 1), which is not consistent with this type of artefact.

**Table 1:**
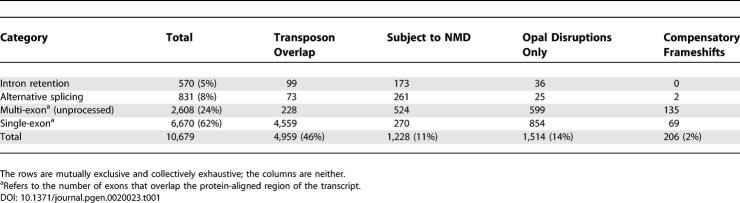
ψmRNA Categories

A further potential source of error is that some Swiss-Prot proteins may be erroneous translations of frameshifted or noncoding nucleotide sequences. In fact, this is why we used only the manually curated Swiss-Prot database, since we frequently encountered this sort of error when using the more extensive TrEMBL set. In addition, we excluded proteins with the keyword “hypothetical protein”, which is defined as “predicted protein for which there is no experimental evidence that it is expressed in vivo”. Nevertheless, the remaining proteins are likely to include a small percentage of errors.

As a final filter, we eliminated cDNAs that map to the mitochondrial genome and whose only disruptions are internal TGA stop codons, since TGA encodes tryptophan in the mitochondrion. As might be expected, there are no mitochondrial ψmRNAs after applying this criterion.

### Number of ψmRNAs

**Figure 1:**
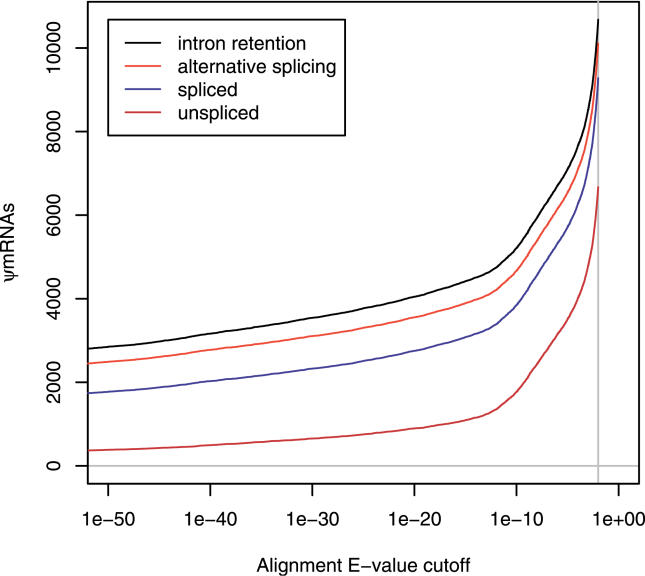
Number of ψmRNAs as a Function of Alignment *E*-Value Cutoff

The pipeline described above indicates that 10,679 out of 102,801 FANTOM3 cDNAs are ψmRNAs (Table S1; format described at http://song.sourceforge.net/gff3.shtml). Since we used an arbitrary *E*-value threshold of 0.01, it is worth examining how the number of ψmRNA predictions varies with *E*-value cutoff. Figure 1 reveals a discontinuity around *E* = 10^−12^. There are 4,746 ψmRNAs with *E* ≤ 10^−12^, the bulk of which are multi-exon (unprocessed), as might be expected since unprocessed pseudogenes are more likely than processed pseudogenes to possess upstream promoters and be transcribed, owing to the different mechanisms by which they are formed. At *E* > 10^−12^ there is a rapid increase in the number of single-exon ψmRNAs, most of which overlap transposon sequences (Table 1). These transposon-associated predictions are discussed further below. Clearly, the number of ψmRNAs we report depends sensitively on the *E*-value cutoff. This is expected because ψmRNAs have diverged from their protein-coding homologues by varying degrees, and there comes a point where the sequence similarity is not statistically significant. It is more accurate to say that the number of *detectable* ψmRNAs is around 10,000. Finally, of course, neither the FANTOM3 transcriptome set nor the reference Swiss-Prot set is complete, so almost certainly additional ψmRNAs remain to be discovered, e.g., we will miss ψmRNAs that are not similar to any known protein.

### Redundancy

The FANTOM collection includes groups of cDNAs that come from the same genomic locus. When ψmRNAs with shared exonic nucleotides are clustered (the transcriptional unit criterion [3]), they reduce to 8,515 clusters. Alternatively, when ψmRNAs with identical genomic mappings of their protein homology segments are clustered, they reduce to 9,583 clusters. cDNAs within such a cluster may differ outside the protein homology region, which could affect their biological behaviour, e.g., a downstream splice site could promote nonsense-mediated decay (NMD). In the following analyses, we either use all 10,679 ψmRNAs or the 9,583 ψmRNA clusters when focusing especially on the protein homology segments. In any case, the ~10,000 ψmRNAs are mostly distinct.

### Categories of ψmRNAs

We sought to understand ψmRNAs better by classifying them based on how they are produced and other basic properties (Table 1). In 570 cases, all the frame disruptions were associated with large unspliced insertions (≥15 nucleotides) in the transcript relative to their homologous proteins: a signature of intron retention. Some of these cases might be transcribed pseudogenes with large insertion mutations, but in all the cases we investigated manually there were alternative transcripts that spliced out the inserted region. Intron retention is difficult to analyse because it is hard to tell whether we have captured incompletely processed pre-mRNA or genuine splice variants. Presumably these 570 cases include some artefactual, immature sequences and some genuine ψmRNAs.

In a further 831 ψmRNAs, all the frame disruptions were associated with either splice junctions or unspliced insertions as above. In these cases the frame disruptions could be avoided by altering the splicing pattern, and in all cases that we investigated manually there were indeed splice variants that avoided the frame disruptions. So these ψmRNAs are disrupted splice variants of protein-coding genes. The disruptions arise in various ways. Frameshifts are caused by the use of out-of-frame alternative donor and acceptor splice sites, or by skipping or inclusion of exons whose length is not a multiple of three. Internal stop codons arise from alternative splice sites that cause extra genomic sequence to be incorporated in the exons, or from inclusion of facultative exons. Since the FANTOM cDNA collection is biased against finding multiple variants of the same gene [20], the proportion of ψmRNAs formed through splice variation is an underestimate.

The remaining ψmRNAs include 2,608 that have splice junctions within the protein-aligned region, and 6,670 that do not. The former presumably derive from unprocessed pseudogenes, whereas the latter may come from processed pseudogenes or single exons of unprocessed pseudogenes. For the majority of single-exon ψmRNAs, the protein homology segment overlaps a transposon sequence. Overall, a large majority of ψmRNAs in this list derive from processed and unprocessed pseudogenes, although this might simply reflect the FANTOM bias against splice variants.

### Popular ψmRNAs and Transposons

Some Swiss-Prot proteins have multiple ψmRNA homologues. Table 2 lists the top ten proteins with the most homologues among the clustered ψmRNAs. For all these proteins, the region of the ψmRNA aligned to the protein usually overlaps a particular type of transposon, indicated in the table. Some of these proteins are components of active LINE elements and endogenous retroviruses: the genome contains numerous inactive, decaying copies of such elements, so it is not too surprising that many transcripts contain disabled homologues of these proteins. These are bona fide pseudogenes. Other cases appear to have a converse history, where ancestrally noncoding sequences have been incorporated into a protein-coding region. For example, the mouse *Jak3* gene has one splice variant that incorporates an Alu element within the protein-coding segment, and the second coding exon of the mouse *Nedd4* gene overlaps a B2 SINE. So these proteins are (genuinely) homologous to many noncoding transcripts that contain Alu and B2 elements. Although it seems strange to call SINE-containing transcripts ψmRNAs, and they can certainly be set aside as a special case, they arguably do fit the definition of a ψmRNA since they contain noncoding sequence that is homologous to protein-coding sequence.

**Table 2:**
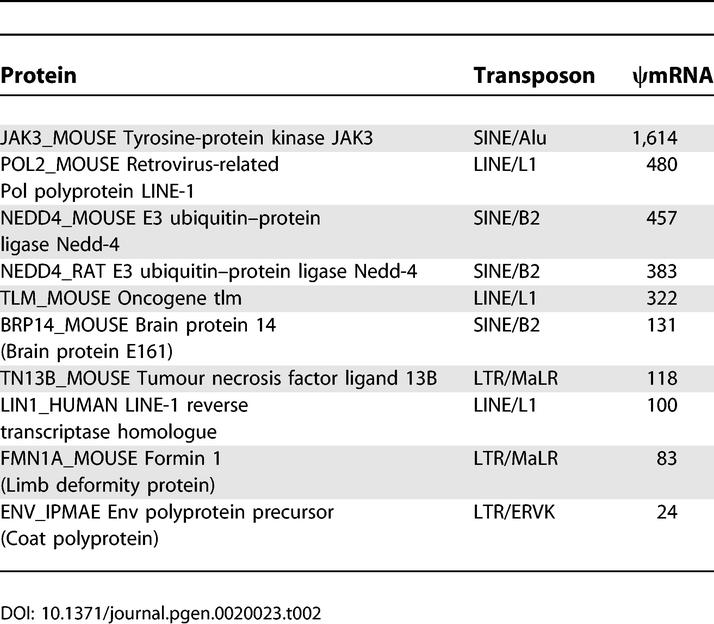
Top Ten Proteins with Most ψmRNA Homologues

We also examined which categories of protein are overrepresented among ψmRNA homologues, compared to FANTOM mRNAs. Swiss-Prot entries include manually curated keywords that categorise the proteins according to functional, structural, and other criteria. Among the clustered ψmRNAs, 119 are homologous to ribosomal proteins (*p* = 3 × 10^−9^), 206 to G-protein-coupled receptors (*p* = 10^−17^), and 158 to polyproteins (*p* = 4 × 10^−47^). There are many ribosomal protein and G-protein-coupled receptor pseudogenes [21,22], and so their overrepresentation among ψmRNAs is not surprising. Polyproteins are cleaved to produce several functional polypeptides: their overrepresentation here suggests that some ψmRNAs may encode truncated polyproteins that generate a subset of the polypeptides.

### Potential Truncated Proteins and NMD

Transcripts with reading frame disruptions may be translatable into partial protein sequences. For example, the section between the start codon (if it is present) and the first frame disruption might be translated. If the first disruption is a frameshift, a stop codon will usually follow soon after since on average three out of 64 codons are stops in noncoding frames. In some cases there are frame disruptions near the beginning of the transcript–protein alignment, followed by a long, undisrupted region at the 3′ end with an alternative or internal in-frame start codon. In these cases it is tempting to speculate that the long 3′ region is translated, although if the start codon is present it is perhaps more plausible that a short peptide is translated from the start of the aligned region. We considered both possibilities for the ψmRNA predictions. We emphasise that our ψmRNA set does not include every splice variant that encodes a truncated protein: it includes such splice variants only if they contain out-of-frame protein-coding sequence.

**Figure 2:**
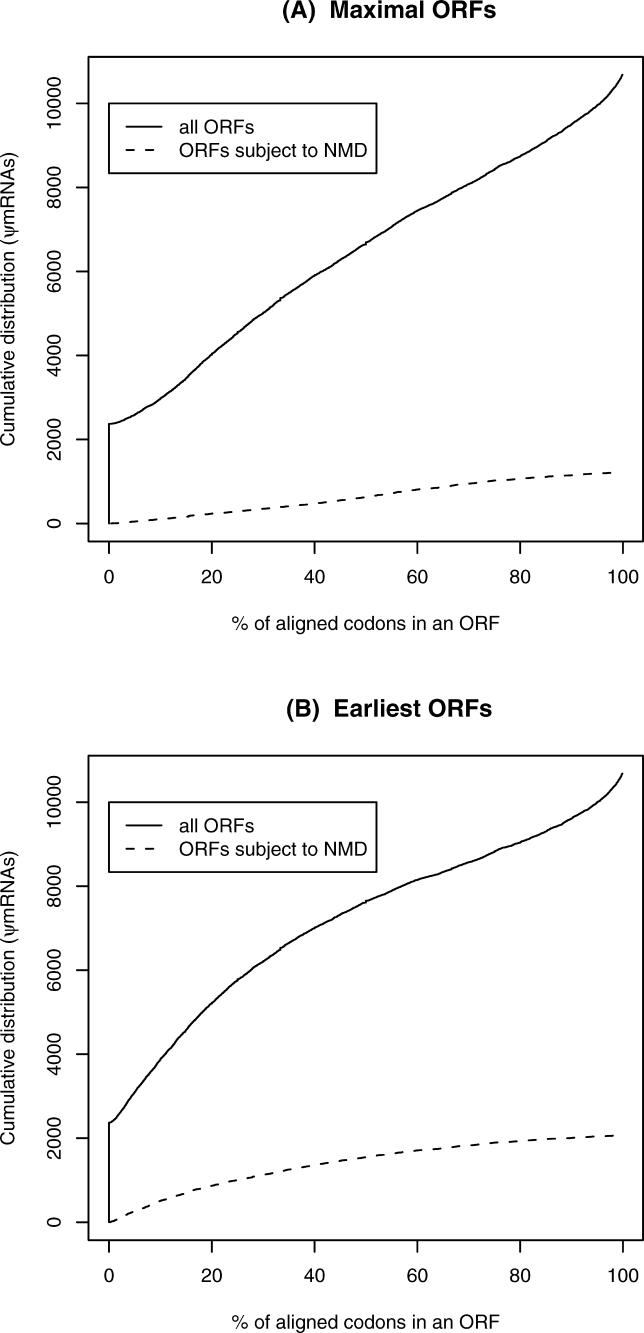
Truncated ORFs of ψmRNAs and Potential for NMD We chose either the ORF with the maximal number of codons (A) or the ORF whose in-frame overlap is earliest (B).

We identified translatable open reading frames (ORFs) in the ψmRNAs that have some in-frame overlap with the protein-aligned region. Sometimes more than one such ORF is present. We chose either the ORF with the maximal number of codons aligned in-frame to protein residues (Figure 2A), or the ORF whose in-frame overlap is earliest (most upstream) (Figure 2B). Almost a quarter of ψmRNAs (2,372) have no ORF with in-frame overlap with the transcript–protein alignment. On the other hand, about a third of predicted ψmRNAs (3,557) have ORFs of 100 aa or more that overlap some protein residues in-frame, and for approximately 10% of predicted ψmRNAs, the ORF covers greater than 90% of the aligned protein (1,178 ψmRNAs using maximal ORFs and 1,069 ψmRNAs using earliest ORFs). These mostly translatable cases may well encode functional proteins, representing benign protein evolution by slight changes in the start or end of translation. There are several possibilities for the intermediate cases: they may encode functional, truncated proteins such as dominant negatives, they may be untranslated, or they may undergo accidental translation into non-functional proteins.

NMD is a phenomenon whereby mRNA molecules with nonsense (premature termination) codons undergo rapid degradation. However, the stop codons must lie at least 50–55 nt upstream of an intron. Interestingly, it has been proposed that NMD has a universal role in proofreading mRNA, protecting the cell against potentially toxic dominant negative truncated proteins. Such dominant negatives could arise for example when a ligand-binding domain is preserved but the signal transduction domain is absent [23]. It has also been shown that in the absence of NMD, the cell overexpresses transcripts arising from retroviral and retroposed elements [24]. The FANTOM dataset is expected to contain few NMD transcripts, as these are rapidly degraded and, therefore, not likely to be selected for during cloning and full-length sequencing. We assessed how many ψmRNAs may be subject to NMD by checking for premature stop codons 55 nt or more upstream of a splice junction. Supposing that either the maximal ORFs or earliest ORFs are utilised, a fairly small minority of ψmRNAs appear subject to NMD: 1,228 and 2,077, respectively (Figure 2). In fact, the maximal and earliest ORFs are distinct in only 2,185 cases; in these cases 1,042 earliest ORFs and 193 maximal ORFs satisfy the NMD criterion. This large discrepancy suggests that if a ψmRNA is translated, the maximal ORF is more likely to be utilised.

### Potential Selenoproteins

The opal codon TGA usually encodes a translation stop but occasionally encodes the rare amino acid selenocysteine. It has been reported that the human selenoproteome consists of 25 selenoproteins [25]. Thus, ψmRNA predictions that have internal TGA codons as their only disruptions might actually encode selenoproteins.

**Figure 3:**
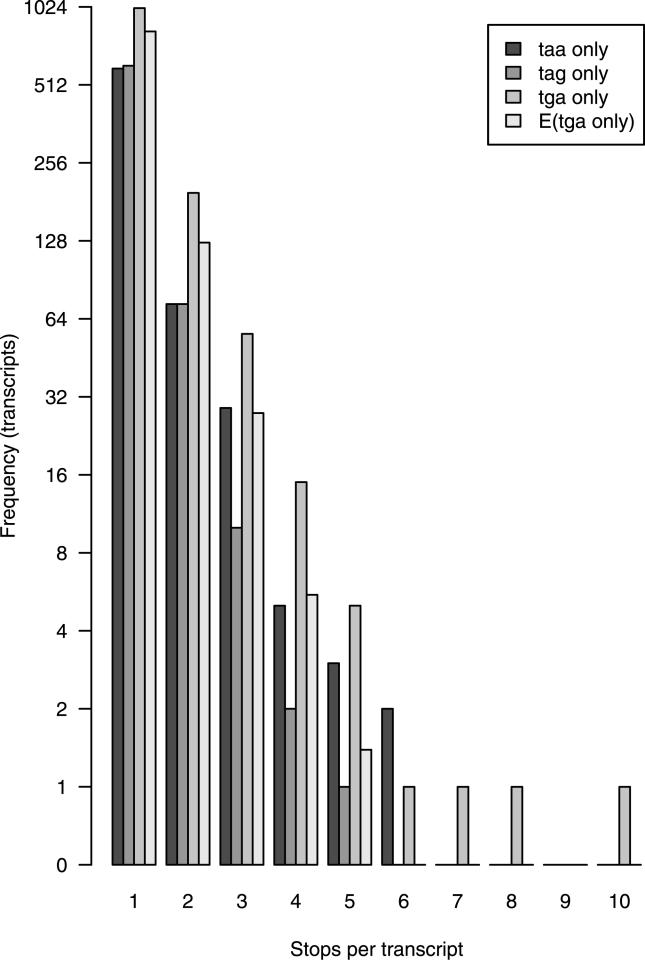
An Excess Number of ψmRNAs Have TGA Stop Codons as the Only Reading Frame Disruption

To assess the impact of selenoproteins on our dataset, we plotted the number of ψmRNA predictions from the clustered set that have varying numbers of internal TGA codons exclusively and no other reading frame disruptions (Figure 3). As a control, we also plotted numbers of ψmRNAs with exclusively TAA or TAG disruptions. There are consistently more cDNAs with TGA disruptions only than TAA only or TAG only: often 2-fold more. As a further control, we counted numbers of internal TAA, TAG, and TGA codons in ψmRNAs with more than one type of internal stop codon (but no frameshifts). These results (2,463 TAA, 2,107 TAG, 2,709 TGA) were used to estimate the expected numbers of TGA-only ψmRNAs, assuming internal stop codons occur randomly and independently in these proportions. These expected numbers are always less than the observed numbers (Figure 3), and in total there are around 300 more TGA-only cases than expected.

The most extreme case has ten internal TGA codons and no other frame disruptions (Figure 3), a very unlikely occurrence if the three types of stop codon are utilised randomly and independently. In fact, this gene encodes a well-characterised selenoprotein, selenoprotein P, thought to function in selenium homeostasis and oxidant defence. Twenty other cases align to known selenoproteins, each with one TGA codon. The two cases with seven and eight TGA codons have borderline FASTX *E*-values, and might represent novel selenoproteins. An alternative explanation of repeated stop codons, tandem repeats, is unlikely since tandem repeats were filtered using XNU. These results suggest that mice, and by extension perhaps humans, possess many more than 25 selenoproteins.

Selenoproteins often contain a particular secondary structure (SECIS) in the 3′ UTR, so we used the program SECISearch to search for these in the ψmRNAs [25]. We found no enrichment in predicted SECIS elements in the ψmRNAs with opal codons once we removed RNAs encoding known selenoproteins. The progam has a high false negative rate (28%), so this analysis does not rule out the possibility that many of the TGA-containing ψmRNAs may actually encode novel selenoproteins with the SECIS motif, or that an alternative selenoprotein insertion motif is used.

### Nuclear Mitochondrial Pseudogenes

Nuclear mitochondrial pseudogenes (numts) are disabled copies of mitochondrially encoded genes in the nuclear genome. The difference in genetic code (TGA is a tryptophan codon in the mitochondrion but a stop codon in the nucleus) means that numts are often dead on arrival. The clustered ψmRNA set includes 55 transcribed numts. Two of these, FANTOM clones 9330154B14 and C530050P18, have FASTX *E* < 10^−10^ and are clearly real numts: they align cleanly to nuclear chromosomes but not to the mitochondrial genome. The remainder have FASTX *E* ≥ 0.0001 and are thus borderline cases. Aligning these cDNAs to the mitochondrial genome at the nucleotide level did not clarify the situation: four have BLASTN *E* ≤ 0.01, 13 have *E* ≤ 0.1, and 49 have *E* ≤ 1. They are presumably a mixture of ancient numts and spurious alignments. All but three of these numts have disruptions other than TGA stop codons, so they do not explain the excess TGA-only cases observed in Figure 3.

### Compensatory Frameshifts

**Figure 4:**
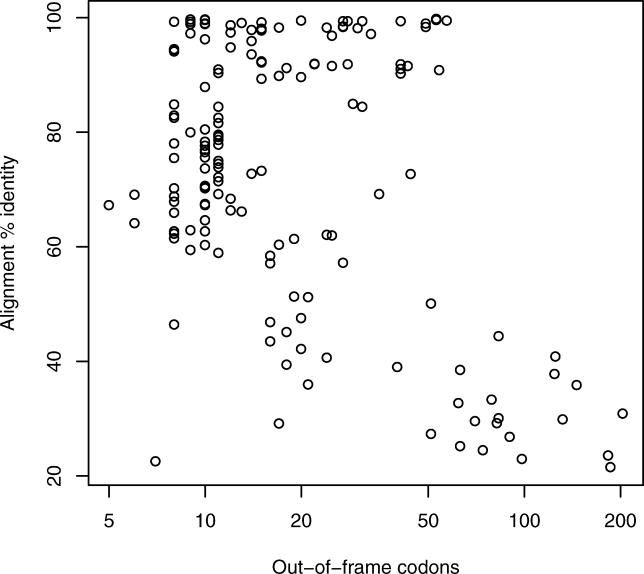
Number of Out-of-Frame Codons in Transcripts with Compensatory Frameshifts

The clustered ψmRNA predictions include 159 cases with compensatory frameshifts. In these cases the transcript–protein alignment undergoes multiple frameshifts but ends up in the same frame in which it started, and the transcript is translatable in this frame without internal stop codons. These cases should arguably be set aside from the ψmRNA list since they may well encode full-length proteins. However, these transcripts are interesting because their frameshifted portions would encode amino acid sequences completely different from their Swiss-Prot homologues. Manual inspection revealed that the frameshifts sometimes occur in low-similarity regions of the alignment, and are thus unreliable, but other alignments are unambiguous because they have close to 100% identity across their entire length. The number of out-of-frame codons is usually less than 20, but the largest reliable case has 57 out-of-frame codons (Figure 4). Our method imposes an artificial lower bound on the number of out-of-frame codons, because the alignment score penalty incurred by two frameshifts cannot be compensated by a short run of intervening matches. Although we have ruled out sequencing errors in the FANTOM cDNAs, compensatory frameshifts might be attributed to sequencing errors in the transcript from which the homologous Swiss-Prot protein was derived. Compensatory frameshifts may be a source of large- and small-scale changes in protein evolution.

The compensatory frameshifts are almost always caused by insertion and deletion mutations, but in at least two cases they arise from alternative splicing (see Discussion). In these cases one section of protein-coding sequence simultaneously encodes two different amino acid sequences in alternative reading frames.

### Evolution of Expressed Pseudogenes

**Figure 5:**
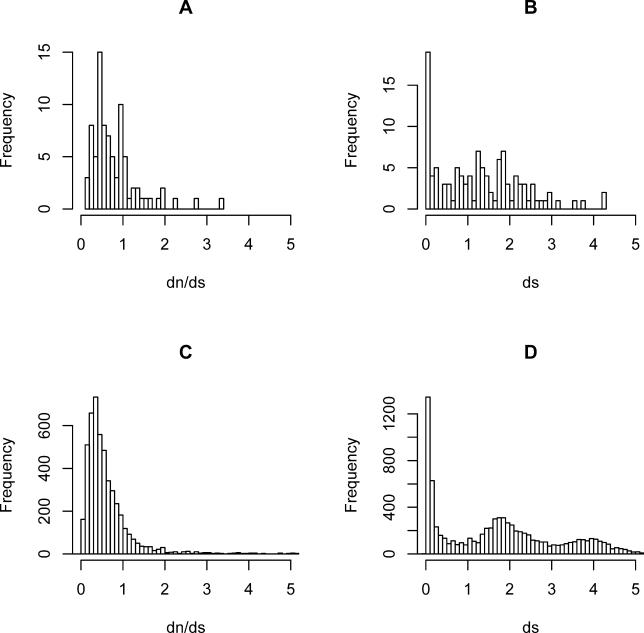
Ratios of Synonymous and Nonsynonymous Substitution in Expressed Pseudogenes (A and B) Distribution of *d*_n_ /*d*_s_ (A) and *d*_s_ (B) for murine expressed pseudogenes. (C and D) Distribution of *d*_n_ /*d*_s_ (C) and *d*_s_ (D) for non-redundant pairs of paralogous mouse protein-coding genes (youngest duplicate chosen for each gene). The *d*_n_/*d*_s_ distribution of murine expressed pseudogenes suggests the existence of two subpopulations: one that has experienced protein-coding constraint and one that has not.

We examined the evolutionary forces acting on expressed pseudogenes by estimating nonsynonymous and synonymous substitution ratios (*d*_n_ and *d*_s_) for a subset of 132 ψmRNAs. As expected, these sequences exhibit a shift towards neutral evolution (towards a *d*_n_/*d*_s_ value of one) in comparison with a control set of mouse protein-coding genes, although the distribution of *d*_n_/*d*_s_ values is heterogeneous (Figure 5). Thus, expressed pseudogenes have a mean *d*_n_/*d*_s_ of 0.809 versus 0.603 for the control set for pairs with *d*_n_ < 2 and *d*_s_ < 2 (Wilcoxon rank sum test, *p* = 3.13 × 10^−6^), or 0.839 and 0.53, respectively, for all pairs (*p* = 4.139 × 10^−10^). Naturally, *d*_n_/*d*_s_ describes only the mode of coding sequence change, and lack of protein-coding conservation does not preclude the possibility of function as noncoding RNA. The distribution of *d*_s_ values for the expressed pseudogenes is similar to that for the control set of paralogues (Figure 5), with a high proportion of pairs with *d*_s_ < 0.2 and a long tail of pairs with higher *d*_s_ values. Assuming the molecular clock, i.e., a similar linear relationship between time and *d*_s_, this suggests that expressed pseudogenes persist in the genome over long evolutionary timescales, similarly to protein-coding paralogues.

**Figure 6:**
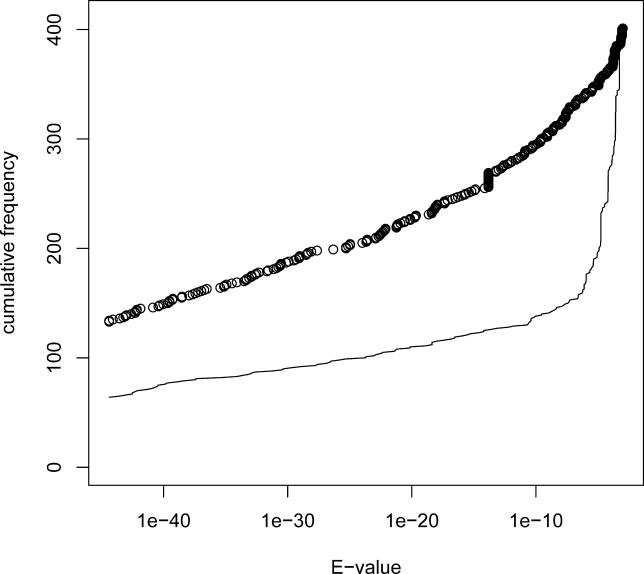
Similarity between Mouse Expressed Pseudogenes and Human Genomic Sequences BLAST *E*-values are shown for murine expressed pseudogenes (line) and paired Swiss-Prot sequences (circles) versus human genome contigs. The total number of reported alignments is similar for both datasets; however, Swiss-Prot entries have consistently twice as many alignments with low *E*-values (0 < *E* < 10^−10^), and with *E* = 0, than do expressed pseudogenes. Alignments with *E* = 0 (132 and 63, respectively) are not visualised as points on the plot, but their respective quantities can be deduced from the shifts of both curves at the root.

To establish the degree of human/mouse conservation, we searched for human orthologues of murine expressed pseudogenes using BLAST. As a positive control, and to differentiate from alignments generated by family members, the Swiss-Prot entries paired with the expressed pseudogenes were used. Figure 6 shows an overlay cumulative plot of *E*-values obtained for FANTOM clones (line) and paired Swiss-Prot sequences (circles). Both the expressed pseudogenes and the paired Swiss-Prot sequences have a similar total number of alignments (395 and 401, respectively), suggesting that non-specific alignments with family members were reported. However, as is evident from Figure 6, at lower *E*-values the cumulative curves strongly diverge, implying that differentiation between the pseudo-orthologue and intact orthologue is feasible. Thus, 55 out of 88 expressed pseudogenes with reported alignments had the lowest *E*-value alignment on a different human contig than did the corresponding Swiss-Prot entry. These FANTOM clones were designated as putatively conserved expressed pseudogenes. This implies significant overall conservation of at least 39% (51 out of 132 expressed pseudogenes). As expected, putative conserved pairs were older than than the remaining set of expressed pseudogenes (mean *d*_s_ of 1.68 versus 1.01, Wilcoxon rank sum test, *p* = 0.002). They also had a lower mean *d*_n_/*d*_s_ of 0.62 versus 0.99 (Wilcoxon rank sum test, *p* = 0.039). However, *d*_n_/*d*_s_ also decreases with age in the control set of paralogues, with means of 0.53 and 0.65 in matched age groups.

### Expression and Promoter Characteristics of ψmRNAs

The locations, shapes, and expression patterns of mouse promoters have been revealed by massive sequencing of CAGE tags (approximately 20-nt sequence tags from the 5′ ends of transcripts) (P. Carninci, A. Sandelin, B. Lenhard, S. Katayama, K. Shimokawa, et al., unpublished data). The overall expression levels of ψmRNAs, measured by numbers of associated CAGE tags, are not significantly higher or lower than those of non-ψmRNA FANTOM transcripts. ψmRNAs are significantly (*p* = 7 × 10^−7^) associated with the BR shape class of promoters, which initiate transcription over a broad region and tend to overlap CpG islands (P. Carninci, A. Sandelin, B. Lenhard, S. Katayama, K. Shimokawa, et al., unpublished data). They are also significantly (*p* = 0.0006) associated with the regionally biased expression class of promoters, where sub-regions of the promoter have distinct tissue specificities (H. Kawaji, S. Katayama, A. Sandelin, C. Kai, J. Kawai, et al., unpublished data). ψmRNA promoters have a significant enrichment relative to other promoters for at least 50 transcription-factor-binding motifs from the TRANSFAC database, associated with 39 transcription factors (Table S2). A large fraction of these transcription factors are nerve system specific and pancreatic beta cell specific (Table S3). Thus ψmRNAs have distinctive promoter and expression patterns, suggesting that they may occupy specific functional niches.

## Discussion

**Figure 7:**
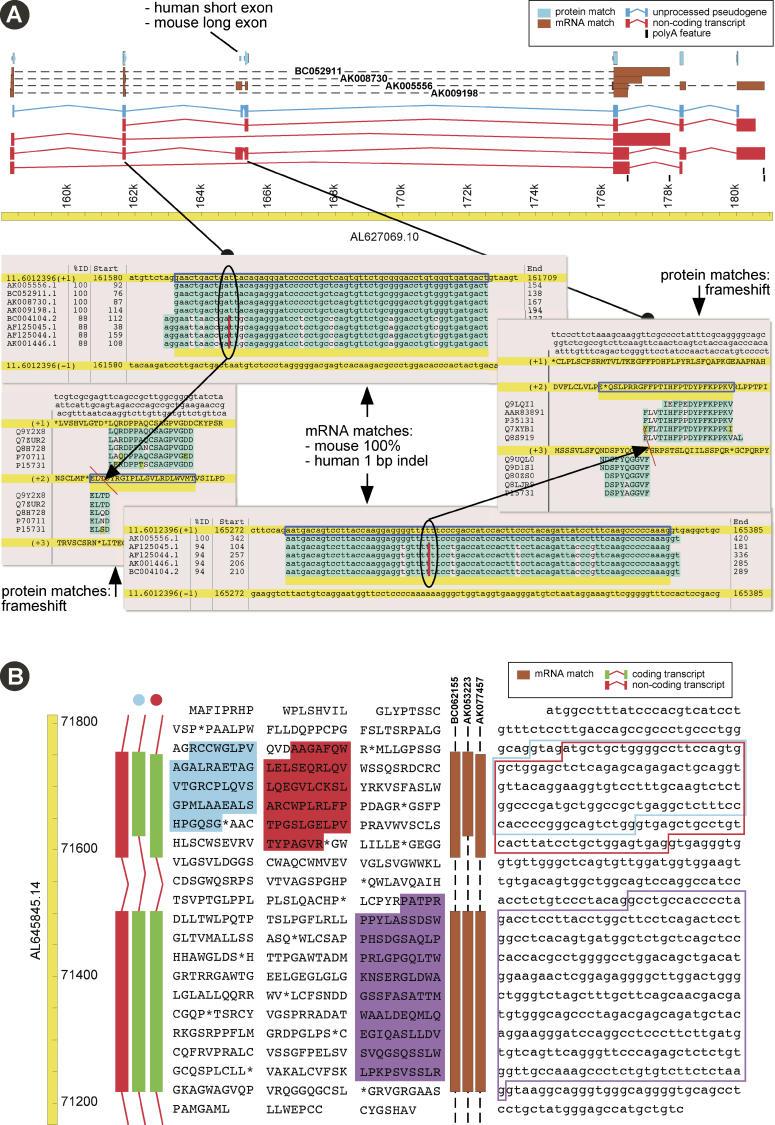
ψmRNA Examples (A) Ubiquitin-conjugating enzyme E2D pseudogene and ψmRNAs on mouse Chromosome 11. Protein homologies (blue track at top) of human *UBE2D* and mouse *Ube2* paralogues to mouse genomic sequence indicate an unprocessed pseudogene (two frameshifts, see alignment panels, blue gene object). Mouse FANTOM mRNAs (brown tracks, with accession numbers) match genomic sequence without disruption (see alignment panels). No current mouse cDNAs or ESTs support the precise exon structure equivalent to the expressed human *UBE2D* gene: for example exon three (indicated) is larger than in the human orthologue. FANTOM mRNAs support two splice variants (red gene objects, numbers two and three from top). Two remaining splice variants are based on mouse EST matches (not shown). Pictures are from AceDB F-map (top) and Blixem (alignments, bottom), modified for clarity. (B) Compensatory frameshifts in FANTOM clone E030045F20 (AK053223) (brown track on the right) on mouse Chromosome 11, caused by splice variation in a gene homologous to human *C22 orf3* at both the 5′ and 3′ end of an internal exon on mouse Chromosome 11. The more common variant is represented by FANTOM mRNA AK077457. The two transcripts (green gene objects on the left) have a different translation for the top exon shown here (blue and red highlights on the translations, for the objects marked with blue and red dots, respectively). Outlines on the DNA sequence show the respective exon boundaries and splice sites. The second exon shown is translated in the same reading frame for both variants (purple highlight), as are preceding and further downstream exons (not shown). A third variant, FANTOM mRNA BC062155, is a non-translating ψmRNA, as it has an out-of-frame alternative splice acceptor but lacks a compensating out-of-frame alternative splice donor site (red gene object on the left). Pictures are from AceDB F-map, modified for clarity.

The existence of ψmRNA highlights the immense difficulty of inferring the transcriptome from the genome. Current gene prediction methods struggle to identify standard protein-coding genes and are often confounded by pseudogenes; ψmRNA is utterly beyond them. A deeper issue is that ψmRNA does not necessarily correspond exactly to identifiable genomic entities. For example Figure 7A shows a ubiquitin-conjugating enzyme E2D pseudogene on mouse Chromosome 11, defined by protein homology, along with several ψmRNA transcripts from this locus. The pseudogene and the transcripts contain frameshifts that prevent translation; the syntenic region in human lacks the frameshifts and encodes a functional protein. Remarkably, none of the transcripts exactly match the exon–intron structure of the pseudogene. The bottom-most transcript in the figure skips both frameshifted exons, but its first two exons are joined out-of-frame: it would be classified as an alternative-splicing-induced ψmRNA by the criteria used in this study. This illustrates that ψmRNAs are not exactly transcribed pseudogenes, but a novel kind of purely transcriptomic object.

Alternative-splicing-induced compensatory frameshifts are another example of a particularly complex relationship between genome and transcriptome (Figure 7B). The highlighted transcript uses an alternative acceptor splice site 4 nt upstream of the canonical acceptor, inducing a frameshift. The subsequent donor splice site is 34 nt upstream of the canonical donor, restoring the reading frame in the next exon. The illustrated exon is translatable in both frames without stop codons. The alternative splice sites are supported by multiple cDNAs and expressed sequence tags (ESTs), so if they are “splicing noise” they are consistent noise. There is no reason to doubt that the frameshifted variant would be translated, and since much of its sequence is identical, it may share many of the canonical protein's interaction partners and interfere with its function.

Full-length sequencing shows that ψmRNAs are expressed, and alignments to the genome show how they are generated. Further studies are required to address the functional relevance and biological role of these transcripts. In the long-term perspective, carefully designed custom oligonucleotide arrays targeted against discriminating features (often located in the 3′ or 5′ UTRs), quantitative real-time PCR, or high throughput RNA-level single-nucleotide-polymorphism-like assays (targeting a stop codon, or a frameshift in ψmRNA) could be used to measure relative expression of ψmRNAs and intact paralogues.

A significant advantage of this study is that the dataset that is used to infer expression (the FANTOM3 full-length cDNA collection), also provides information about the structure and identity of the ψmRNAs. Additional confirmation is achieved though the well-integrated in-house CAGE dataset. Mapping to external sources of expression data, such as microarrays or ESTs, introduces risk of misassignment of probes/tags due to differences in experimental protocols, data post-processing, database formats, or inconsistent gene and sample annotation practices. Additionally, microarrays are prone to cross-hybridization, especially when probes target members of multi-gene families. For example, we have previously found that when a pair of human or mouse paralogues are mapped to Affymetrix probes with name suffixes _f_at, “sequence family”, and _s_at, “similarity constraint” (see Affymetrix manual, Data Analysis Fundamentals, Appendix B), expression similarity calculated as a Pearson *R* correlation coefficient is significantly higher than the same value for pairs mapped to probes with normal tiling [26].

In conclusion, the transcriptome can no longer be regarded as a simple mirror of the genome, or a redundant layer between genome and proteome. This was already indicated by the high incidence of alternative splicing, but the non-standard transcripts surveyed here provide even more compelling examples. The recently developed field of systems biology stresses the complex emergent behaviour that lies between genotype and phenotype: this complexity begins with the transcriptome.

## Materials and Methods

### Transcript–protein alignments.

The FANTOM3 cDNA sequences were aligned to the proteins in Swiss-Prot release 46.4 using FASTX version 3.4t25 with options –Q –E 0.01 –m9c –H −3 –s BL62 –S –t t –f 11. Prior to alignment, low-complexity protein sequences were soft-masked using pseg –z 1 –q as recommended in the FASTA documentation [27]. Despite this masking, we observed spurious alignments involving tandem repeats. Therefore, the proteins were also soft-masked using xnu –n 0 −60 –o [28], and the union of pseg and xnu masking was applied.

Our experiences with other alignment methods and parameters may be informative. We initially tried FASTY, which allows frameshifts within rather than between codons and claims to produce better alignments than FASTX. However, we found that FASTY hinders identification of frameshifts caused by intron retention and alternative splicing: it often places frameshifts one codon away from alignment gaps caused by these phenomena rather than directly adjacent to the gaps. We also tried NCBI BLASTX (2004–12–05 snapshot) with the –w option to allow frameshifts, but a bug causes garbled output for some input combinations. On the other hand, the default scoring scheme used by BLASTX is superior to that used by FASTX: the FASTX alignments tended to extend more aggressively from a reliable core region into low-similarity regions with spurious frameshifts and aligned stop codons. We therefore used the more stringent BLASTX parameters (BLOSUM62 matrix and gap opening penalty) for FASTX. WUBLAST does not appear to have a frameshifting alignment option.

### Transcript–genome alignments.

Alignments of the cDNAs to version mm5 of the mouse genome were obtained from ftp://fantom.gsc.riken.jp/FANTOM3/mapping_materials/f3_mm5_best.gff.gz.

### Association of frame disruptions with intron retention and splicing.

The transcript–protein alignments were first converted to “double gap” format, i.e., an alternating series of ungapped aligned segments separated by unaligned segments in either or both sequences. In this representation frameshifts are unaligned segments of the cDNA with length not divisible by three (possibly having length −1 for reverse frameshifts). Frameshifts with splice junctions within them or less than one codon distant (since FASTX constrains frameshifts to lie between codons) were classed as splice-associated. Frameshifts larger than 15 nt that were not splice-associated were classed as intron-associated. In-frame stop codons lying within unaligned segments of the cDNA with length divisible by three were classed as splice-associated if there was a splice junction within the unaligned segment or less than one codon distant, or intron-associated if they were not splice-associated and the unaligned segment was 15 nt or greater. These criteria rely on the alignment being very accurate, and so some associations of frame disruptions with intron retention and splicing are missed.

### Transposon identification.

Transposon sequences were identified in the cDNAs using version 2002/05/15 of RepeatMasker with options –mus –xm –xsmall –a [29]. Only repeats of class SINE, LINE, LTR, and DNA were considered.

### Overrepresentation of protein categories.

Overrepresented protein categories were detected in a foreground set (ψmRNAs) relative to a background set (all FANTOM cDNAs with Swiss-Prot homologues). The background set was constructed by keeping the top Swiss-Prot hit to each cDNA regardless of reading frame disruptions (but excluding hypothetical proteins). Each set was made non-redundant by clustering sequences with identical genomic mappings of their protein homology segments. Sequences homologous to the top ten proteins in Table 2 were ignored, to prevent them from dominating the result. Finally, Swiss-Prot keywords were counted in the foreground and background sets, and the probability of the overrepresentation arising by chance was calculated using the hypergeometric distribution for sampling without replacement.

### Evolutionary analysis.

FANTOM clones aligned to a mouse Swiss-Prot protein were selected from the total dataset of transcript–protein alignments. We selected for stops rather then frameshifts (as coding sites have to be aligned properly for *d*_n_ and *d*_s_ calculations to be meaningful). Expressed pseudogenes rather than intron retention or disrupted splice variants were selected (cases where a FANTOM stop codon faces a gap in the Swiss-Prot sequence were omitted). Most Swiss-Prot sequences paired with multiple FANTOM clones were found to contain signatures of retro-elements (RepeatMasker) and were not included in further analysis. This procedure resulted in a set of 132 FANTOM clones mapping uniquely to Swiss-Prot entries, termed murine expressed pseudogenes, which were used to estimate synonymous and nonsynonymous substitution ratios and the degree of human/mouse conservation. Nucleotide sequences corresponding to the Swiss-Prot proteins were fetched from the EMBL database. EMBL and FANTOM clones were then aligned using protein alignment (CLUSTALW with default parameters [30]) as guide (custom scripts). Non-redundant pairs (8,840) of mouse paralogues predicted by Ensembl were used as a control dataset of normal gene duplicates [31]. Pairwise *d*_n_ and *d*_s_ distances were calculated using the method of Young and Nielsen implemented in the program Yn00 from the PAML suite (version 3.13) [32]. Sequences with *d*_n_ or *d*_s_ equal to zero or incalculable were ignored, as *d*_n_/*d*_s_ ratios for these cases are not meaningful or cannot be calculated (20 out of 132 murine expressed pseudogenes). A ceiling for *d*_n_ and *d*_s_ saturation was set at two when calculating *d*_n_/*d*_s_, with the exception of comparison of putatively conserved versus remaining expressed pseudogenes, where no such ceiling was used to maximise the number of available datapoints (83 datapoints with the ceiling, 112 without).

NCBI BLAST2 was used to search for human orthologues of murine expressed pseudogenes [33]. Repeat-masked human genome contigs (26,881 sequences) were downloaded from the Ensembl ftp site as a multiple FASTA file, and converted to a BLAST searchable database using FORMATDB. The BLASTN filtering option was disabled and the E-value cutoff was set at 0.001. Output was set to tabular using the –m 8 option and uploaded to a MySQL database for subsequent analysis. Statistics were performed using R.

### Promoter analysis.

The numbers of CAGE tags from promoters of ψmRNA and promoters of non-ψmRNA were compared using the Wilcoxon rank sum test with continuity correction. Fisher's exact test was used to determine enrichment of promoter classes among ψmRNA promoters relative to other promoters. The motif analysis is described in Protocol S1.

## Supporting Information

Protocol S1 Promoter Characteristics (20 KB DOC)

Table S1 10,679 ψmRNAs among the FANTOM cDNA Collection (gff Format) (1.5 MB TXT)

Table S2 Top 50 Promoter Elements Found in the Target Set as Compared to the Background Set of Approximately 40,000 Mouse Promoters (28 KB DOC)

Table S3 Distribution of Transcription Factors from the Top 50 Ranked Promoter Elements across Nine Groups of Transcription Factors (66 KB DOC)

## Abbreviations

ψmRNA: pseudo–messenger RNA
EST: expressed sequence tag
NMD: nonsense-mediated decay
numt: nuclear mitochondrial pseudogene
ORF: open reading frame
