# Supplementary material for: Pseudo–Messenger RNA: Phantoms of the Transcriptome
Source: PLoS Genet. 2006 Apr 28;2(4):e23. doi: 10.1371/journal.pgen.0020023 (PMC1449882; doi:10.1371/journal.pgen.0020023)
Supplement: Protocol S1 — (20 KB DOC) [file pgen.0020023.sd001.doc]

# Promoter characteristics

From tag clusters (TC), we determined a total of 736,403 promoter sequences. There were 8,953 unique TC IDs obtained from <https://genomec.gsc.riken.jp/fantom30/fantom3/cDNA/tc_tissue_specificity/obsolete/050601/pseudo2tc2class.tab> and 9,583 unique transcription start sites of ψmRNA based on <https://genomec.gsc.riken.jp/fantom30/fantom3/cDNA/pseudo/satellite/f3_mm5_pseudo_redundant_ids>. The total number of ψmRNA promoter sequences found by intersecting these two lists was 8,656.

We analysed these 8,656 promoters of ψmRNA, and contrasted their promoter content with ~40,000 randomly collected promoters from the mouse genome. We analysed the [-1,000,+200] regions relative to their transcription start sites. Promoters that had 5% or more ambiguous nucleotides within their sequences were excluded. The target set of promoters of ψmRNA was thereby reduced to 8395, while the background set was reduced to 38,520 promoters.

Then, all available matrix models of TFBSs contained in the TRANSFAC Professional (ver. 8.4) database [S1], were mapped to the extracted sequences. We used *minSUM* profiles for thresholds of the matrix models since these contain the optimized threshold values for the core and matrix scores [S2]. The thresholds in *minSUM* are based on optimization that provides the minimum sum of false positive and false negative TFBS predictions. For determination of over-representation of TFBSs found in the target ψmRNA set, we used the method presented in [S3]. All TFBS mapped to ψmRNA promoters were ranked based on their over-representation index (ORI) as defined in [S3]. For ORI = 1 or close to this value, there is no over-representation of the motif in the target promoter group. We also estimated the likelihood of observing these TFBSs in the target set using the background random promoter set as the reference. The p-values were calculated using hypergeometric distribution by perl package HyGe.pm downloadable from: <http://www.cs.huji.ac.il/~bioskill/Ex2/HyGe.pm>. These p-values were subjected to Bonferroni correction for multiplicity testing. If the corrected p-value of the pattern was <0.05, we placed ‘+’sign after the ORI value in Table S2. Table S2 contains only the top 50 ranked patterns that are found in at least 1% of the ψmRNA promoters. The ranking was based on the ORI values. Of these patterns, 38 are found to be with the corrected p-values < 0.05, suggesting that, based on their presence in the background set, they are not expected in the target promoters in the amounts in which they are found.

The top 50 TFBS patterns considered in this analysis bind in total 39 different TFs of which 28 appear in 38 statistically significant patterns. We also examined what is the distribution of these TFs across several classes of TFs. These classes of TFs are from the TRANSFAC Professional ver.8.4 suite and include adipocytes specific, cell-cycle specific, immune-cell specific, liver specific, lung specific, mucle specific, nerve system specific, pancreatic beta cell specific and pituitary gland specific TF groups. The results are given in Table S3. Among the nine different TF groups considered, we observe a significant participation of TFs that are nerve system specific and pancreatic beta cell specific.

# REFERENCES

[S1] Matys V, Fricke E, Geffers R, Gossling E, Haubrock M, Hehl R, Hornischer K, Karas D, Kel AE, Kel-Margoulis OV, Kloos DU, Land S, Lewicki-Potapov B, Michael H, Munch R, Reuter I, Rotert S, Saxel H, Scheer M, Thiele S, Wingender E (2003) TRANSFAC: transcriptional regulation, from patterns to profiles, Nucleic Acids Res. 31(1):374-8.

[S2] Kel AE, Gossling E, Reuter I, Cheremushkin E, Kel-Margoulis OV, Wingender E (2003) MATCH: A tool for searching transcription factor binding sites in DNA sequences. Nucleic Acids Res. 31(13):3576-9.

[S3] Bajic VB, Choudhary V, Hock CK (2004) Content analysis of the core promoter region of human genes, In Silico Biology*,* (electronic version 4, 0011, 2003), 4:109-125.
