# Supplementary material for: Pseudo–Messenger RNA: Phantoms of the Transcriptome
Source: PLoS Genet. 2006 Apr 28;2(4):e23. doi: 10.1371/journal.pgen.0020023 (PMC1449882; doi:10.1371/journal.pgen.0020023)
Supplement: Table S2 — (28 KB DOC) [file pgen.0020023.st002.doc]

# **Table S2.** Top 50 promoter elements found in the target set as compared to the background set of ~40000 mouse promoters. The Bonferroni corrected p-value based on right-sided Fisher’s exact test is presented in the last column. The significance level of 0.05 is adopted and the significant patterns are denoted by + sign next to ORI value. The columns represent: promoter element description including strand and TFBS name, ORI, % of target promoter sequence having promoter element pattern, % of background promoter sequences that have that pattern, probability of finding the pattern in the target set (number of promoter sequences that have pattern divided by the total length of target promoter sequences), probability of finding the pattern in the background set, number of target promoter sequences having the pattern, number of background promoter sequences having the pattern, total number of target promoters, total number of background promoters, corrected p-value.

TFBS pattern ORI % TAR % BCG Prob TARGET Prob BACKGR # TAR # BCG TOT TAR TOT BCG p-value

============ ======== ===== ====== =========== =========== ===== ===== ======= ======= ===========

-1 Imperfect Hogness/Goldberg BOX 5.2564+ 5.55 2.44 4.824e-005 2.085e-005 466 941 8395 38520 3.031e-041

-1 Major T-antigen 4.1763+ 8.66 4.29 7.862e-005 3.799e-005 727 1653 8395 38520 8.962e-051

+1 PAX6 3.0419+ 13.25 8.02 1.631e-004 8.850e-005 1112 3091 8395 38520 1.429e-044

+1 Tal-1beta:E47 2.7825+ 3.99 2.39 3.405e-005 2.042e-005 335 921 8395 38520 5.119e-012

-1 Croc 2.6840+ 13.54 9.25 2.019e-004 1.101e-004 1137 3564 8395 38520 1.988e-027

-1 Freac-3 2.4991 1.16 0.73 9.728e-006 6.166e-006 97 281 8395 38520 1.414e-001

-1 XFD-1 2.4562+ 10.42 6.79 9.946e-005 6.220e-005 875 2614 8395 38520 2.098e-025

-1 FXR/RXR-alpha 2.4344 1.00 0.64 8.438e-006 5.408e-006 84 247 8395 38520 5.764e-001

+1 Tal-1beta:ITF-2 2.3872+ 4.59 2.99 3.971e-005 2.551e-005 385 1152 8395 38520 8.972e-010

+1 Imperfect Hogness/Goldberg BOX 2.2864+ 2.91 1.92 2.472e-005 1.633e-005 244 741 8395 38520 4.184e-005

+1 Bcd 2.2534+ 16.43 11.14 1.532e-004 1.002e-004 1379 4292 8395 38520 1.137e-035

+1 Cart-1 2.1816+ 8.49 5.74 7.604e-005 5.160e-005 713 2210 8395 38520 7.144e-017

+1 POU6F1 2.1546+ 5.57 3.84 4.963e-005 3.345e-005 468 1479 8395 38520 2.937e-009

+1 NKX6-1 2.0836+ 4.13 2.85 3.544e-005 2.464e-005 347 1099 8395 38520 2.670e-006

-1 MCM1+SFF 2.0484+ 17.59 12.71 1.903e-004 1.286e-004 1477 4896 8395 38520 1.136e-027

-1 BR-C Z4 2.0183+ 63.31 53.13 2.460e-003 1.452e-003 5315 20467 8395 38520 1.466e-062

+1 Major T-antigen 1.9974+ 5.24 3.74 4.675e-005 3.282e-005 440 1440 8395 38520 6.501e-007

+1 Tal-1alpha:E47 1.9799+ 5.21 3.72 4.497e-005 3.178e-005 437 1433 8395 38520 9.182e-007

-1 FOXO3 1.9668 1.30 0.94 1.112e-005 7.831e-006 109 361 8395 38520 1.000e+000

-1 POU6F1 1.9510+ 5.35 3.83 4.705e-005 3.366e-005 449 1476 8395 38520 6.942e-007

-1 MADS-A 1.9470+ 3.54 2.53 3.038e-005 2.183e-005 297 974 8395 38520 5.087e-004

+1 MADS-A 1.9396+ 3.03 2.15 2.591e-005 1.876e-005 254 830 8395 38520 2.785e-003

+1 CDP CR3 1.9255 1.33 0.96 1.122e-005 8.091e-006 112 370 8395 38520 1.000e+000

-1 PITX2 1.9216+ 26.61 19.74 2.660e-004 1.867e-004 2234 7602 8395 38520 7.749e-040

-1 CHX10 1.9075 1.57 1.14 1.340e-005 9.649e-006 132 441 8395 38520 1.000e+000

+1 Lhx3 1.8942+ 5.24 3.80 5.072e-005 3.693e-005 440 1464 8395 38520 3.647e-006

-1 Hb 1.8863+ 65.07 55.76 3.684e-003 2.279e-003 5463 21479 8395 38520 4.902e-053

+1 FOXD3 1.8790+ 69.93 61.18 3.254e-003 1.980e-003 5871 23566 8395 38520 2.283e-049

-1 A 1.8724+ 6.28 4.60 5.450e-005 3.976e-005 527 1770 8395 38520 2.886e-007

+1 Freac-3 1.8634 1.00 0.75 8.735e-006 6.274e-006 84 288 8395 38520 1.000e+000

+1 HFH-4 1.8521 2.19 1.60 1.906e-005 1.413e-005 184 615 8395 38520 1.798e-001

-1 Freac-7 1.8334+ 26.48 21.19 3.605e-004 2.457e-004 2223 8164 8395 38520 2.120e-022

-1 Cart-1 1.8291+ 7.68 5.70 6.909e-005 5.090e-005 645 2196 8395 38520 1.715e-008

+1 HFH-3 1.8172+ 31.16 24.98 5.075e-004 3.484e-004 2616 9623 8395 38520 9.412e-028

+1 STAT5A (homodimer) 1.8010+ 3.60 2.69 3.067e-005 2.278e-005 302 1036 8395 38520 8.984e-003

-1 PAX6 1.8009+ 9.82 7.48 1.109e-004 8.080e-005 824 2881 8395 38520 2.003e-009

+1 CF2-II 1.7523+ 26.13 19.98 8.985e-004 6.707e-004 2194 7696 8395 38520 1.031e-031

-1 Athb-1 1.7511 2.17 1.64 1.846e-005 1.391e-005 182 633 8395 38520 9.670e-001

+1 Nrf-1 1.7397 2.10 1.63 1.975e-005 1.462e-005 176 627 8395 38520 1.000e+000

-1 alpha-CP1 1.7323 1.04 0.79 8.934e-006 6.728e-006 87 306 8395 38520 1.000e+000

-1 MADS-B 1.7265+ 10.68 8.18 9.728e-005 7.362e-005 897 3150 8395 38520 3.995e-010

-1 Tal-1beta:E47 1.7247 2.80 2.14 2.392e-005 1.811e-005 235 826 8395 38520 3.002e-001

-1 FOXJ2 1.7171+ 47.49 39.13 8.465e-004 5.984e-004 3987 15072 8395 38520 5.448e-042

-1 Lhx3 1.7121+ 5.25 4.03 5.122e-005 3.901e-005 441 1552 8395 38520 7.496e-004

-1 Brn-2 1.7118+ 29.59 23.16 3.162e-004 2.359e-004 2484 8922 8395 38520 1.860e-031

-1 S8 1.7071+ 4.57 3.53 4.358e-005 3.312e-005 384 1358 8395 38520 5.866e-003

+1 FOX 1.7000+ 49.48 41.78 1.015e-003 7.071e-004 4154 16092 8395 38520 5.317e-035

+1 Ftz 1.6849 1.19 0.91 9.927e-006 7.702e-006 100 351 8395 38520 1.000e+000

+1 XFD-1 1.6684+ 8.77 6.88 8.140e-005 6.218e-005 736 2650 8395 38520 2.664e-006

+1 Brn-2 1.6654+ 27.41 21.60 2.854e-004 2.175e-004 2301 8319 8395 38520 7.583e-027
