# Supplementary material for: Pseudo–Messenger RNA: Phantoms of the Transcriptome
Source: PLoS Genet. 2006 Apr 28;2(4):e23. doi: 10.1371/journal.pgen.0020023 (PMC1449882; doi:10.1371/journal.pgen.0020023)
Supplement: Table S3 — (66 KB DOC) [file pgen.0020023.st003.doc]

**Table S3.** Distribution of TFs from the top 50 ranked promoter elements across nine groups of TFs. Nerve system specific and pancreatic beta cell specific appear to be most abundant groups.

| TFNAME | ADIP | CC | IMMcell | LIV | LUNG | MUSC | NERV | PANC | PITU |
| --- | --- | --- | --- | --- | --- | --- | --- | --- | --- |
| A | 0 | 0 | 0 | 0 | 0 | 0 | 0 | 0 | 0 |
| Athb-1 | 0 | 0 | 0 | 0 | 0 | 0 | 0 | 0 | 0 |
| BR-C Z4 | 0 | 0 | 0 | 0 | 0 | 0 | 0 | 0 | 0 |
| Bcd | 0 | 0 | 0 | 0 | 0 | 0 | 0 | 0 | 0 |
| Brn-2 | 0 | 0 | 0 | 0 | 0 | 0 | 1 | 0 | 0 |
| CDP CR3 | 0 | 0 | 0 | 0 | 0 | 0 | 0 | 0 | 0 |
| CF2-II | 0 | 0 | 0 | 0 | 0 | 0 | 0 | 0 | 0 |
| CHX10 | 0 | 0 | 0 | 0 | 0 | 0 | 1 | 0 | 0 |
| Cart-1 | 0 | 0 | 0 | 0 | 0 | 0 | 0 | 0 | 0 |
| Croc | 0 | 0 | 0 | 0 | 0 | 0 | 0 | 0 | 0 |
| FOX | 0 | 0 | 1 | 0 | 1 | 0 | 1 | 0 | 0 |
| FOXD3 | 0 | 0 | 0 | 0 | 0 | 0 | 1 | 0 | 0 |
| FOXJ2 | 0 | 0 | 0 | 0 | 0 | 0 | 0 | 0 | 0 |
| FOXO3 | 0 | 0 | 0 | 0 | 0 | 0 | 0 | 0 | 0 |
| FXR/RXR-alpha | 0 | 0 | 0 | 1 | 0 | 0 | 0 | 0 | 0 |
| Freac-3 | 0 | 0 | 0 | 0 | 0 | 0 | 1 | 0 | 0 |
| Freac-7 | 0 | 0 | 0 | 0 | 1 | 0 | 0 | 0 | 0 |
| Ftz | 0 | 0 | 0 | 0 | 0 | 0 | 0 | 0 | 0 |
| HFH-3 | 0 | 0 | 0 | 0 | 0 | 0 | 0 | 0 | 0 |
| HFH-4 | 0 | 0 | 0 | 0 | 1 | 0 | 1 | 0 | 0 |
| Hb | 0 | 0 | 0 | 0 | 0 | 0 | 0 | 0 | 0 |
| Imperfect Hogness/Goldberg BOX | 0 | 0 | 0 | 0 | 0 | 0 | 0 | 0 | 0 |
| Lhx3 | 0 | 0 | 0 | 0 | 0 | 0 | 1 | 0 | 1 |
| MADS-A | 0 | 0 | 0 | 0 | 0 | 0 | 0 | 0 | 0 |
| MADS-B | 0 | 0 | 0 | 0 | 0 | 0 | 0 | 0 | 0 |
| MCM1+SFF | 0 | 0 | 0 | 0 | 0 | 0 | 0 | 0 | 0 |
| Major T-antigen | 0 | 0 | 0 | 0 | 0 | 0 | 0 | 0 | 0 |
| NKX6-1 | 0 | 0 | 0 | 0 | 0 | 0 | 0 | 1 | 0 |
| Nrf-1 | 0 | 0 | 0 | 0 | 0 | 0 | 0 | 0 | 0 |
| PAX6 | 0 | 0 | 0 | 0 | 0 | 0 | 1 | 1 | 0 |
| PITX2 | 0 | 0 | 0 | 0 | 0 | 0 | 0 | 0 | 1 |
| POU6F1 | 0 | 0 | 0 | 0 | 0 | 0 | 1 | 0 | 0 |
| S8 | 0 | 0 | 0 | 0 | 0 | 0 | 0 | 0 | 0 |
| STAT5A (homodimer) | 1 | 0 | 0 | 0 | 0 | 0 | 0 | 1 | 0 |
| Tal-1alpha:E47 | 0 | 0 | 1 | 0 | 0 | 1 | 0 | 1 | 0 |
| Tal-1beta:E47 | 0 | 0 | 1 | 0 | 0 | 1 | 0 | 1 | 0 |
| Tal-1beta:ITF-2 | 0 | 0 | 0 | 0 | 0 | 0 | 0 | 0 | 0 |
| XFD-1 | 0 | 0 | 0 | 0 | 0 | 0 | 0 | 0 | 0 |
| alpha-CP1 | 0 | 1 | 0 | 0 | 0 | 1 | 0 | 1 | 0 |
|  | | | | | | |  |  |  |
| TOTAL = 39 | 1 | 1 | 3 | 1 | 3 | 3 | 9 | 6 | 2 |
